# An interpretable nomogram for predicting sleep disturbance risk in maintenance hemodialysis patients

**DOI:** 10.1007/s11255-026-05091-7

**Published:** 2026-03-11

**Authors:** Haixia Zhang, Linsen Jiang, Rong Ni, Peng Qian, Zhi Wang, Weiwei Li

**Affiliations:** Department of Nephrology, The Second Affiliated Hospital of Soochow University, Suzhou, 215004 China

**Keywords:** Sleep–wake disorders, Renal dialysis, Nomograms, Risk assessment, Explainable artificial intelligence

## Abstract

**Background:**

This study aimed to develop and validate an interpretable nomogram to predict the risk of sleep disturbance in maintenance hemodialysis (MHD) patients.

**Methods:**

In this single-center study, 208 MHD patients were enrolled. Sleep quality was assessed using the Pittsburgh Sleep Quality Index (PSQI), with a score > 5 defining the sleep disorder group. Univariate and multivariable logistic regression analyses identified independent predictors, which were used to construct a nomogram. The model’s performance was evaluated by the area under the receiver operating characteristic curve (AUC), calibration plot, and decision curve analysis (DCA). Explainable AI (SHapley Additive exPlanations, SHAP) was applied to interpret the model.

**Results:**

Among 208 patients, 144 (69.2%) had sleep disturbance. Multivariable analysis identified restless legs syndrome (RLS) (OR = 4.52, 95% CI: 2.22–9.18), older age (OR = 1.04, 95% CI: 1.01–1.07), lower serum albumin (Alb) (OR = 0.86, 95% CI: 0.77–0.96), and lower parathyroid hormone (PTH) (OR = 0.99, 95% CI: 0.99–0.99) as independent predictors. Primary kidney disease etiology was retained for clinical comprehensiveness. The nomogram incorporating these five predictors demonstrated good discrimination (AUC = 0.80, 95% CI: 0.73–0.86), satisfactory calibration, and positive net benefit on DCA. SHAP analysis confirmed RLS as the most influential predictor and revealed complex, non-linear relationships for Alb and PTH.

**Conclusion:**

This study presents a validated, interpretable nomogram that is associated with the individual risk of sleep disturbance in MHD patients using five readily available clinical parameters. The tool demonstrates good performance and clinical utility, potentially facilitating early identification and personalized management of high-risk individuals.

## Introduction

Sleep disturbance is a highly prevalent yet often overlooked comorbidity among patients undergoing maintenance hemodialysis (MHD). Studies have reported that over 50–80% of this population experience poor sleep quality, manifesting as difficulties in falling asleep, frequent awakenings, and non-restorative sleep [1, 2]. The etiology of sleep disturbance in MHD patients is multifactorial, encompassing uremic toxin accumulation, electrolyte imbalances, high comorbidity burden, and the psychosocial stress associated with chronic illness and demanding treatment regimens [3].

The consequences of poor sleep extend far beyond daytime fatigue. It is robustly linked to impaired health-related quality of life, increased risks of depression and cognitive decline, and may contribute to the activation of inflammatory pathways and the hypothalamic–pituitary–adrenal axis [4–6]. More critically, sleep disorders have been increasingly recognized as independent risk factors for cardiovascular events and all-cause mortality in the MHD population [7, 8]. Despite its significant impact, sleep disturbance remains underdiagnosed and suboptimally managed in routine clinical practice, partly due to the lack of simple and effective risk assessment tools.

Several factors have been investigated in relation to sleep problems in MHD patients. Restless legs syndrome (RLS) is one of the most well established, with a prevalence substantially higher than in the general population, and it severely fragments sleep architecture [9, 10]. Nutritional status, particularly hypoalbuminemia, as a marker of both inflammation and protein-energy wasting, has also been associated with sleep quality [11, 12]. Furthermore, the roles of mineral bone disease markers like parathyroid hormone (PTH) and the decline in functional capacity with advancing age are areas of growing interest [13–15]. While previous studies have identified numerous potential risk factors, there is a scarcity of research integrating these elements into a practical, clinically applicable predictive model.

Therefore, the primary objective of this study was to develop and validate an interpretable nomogram for predicting the individual risk of sleep disturbance in MHD patients. By incorporating advanced explainable artificial intelligence (XAI) techniques, specifically SHapley Additive exPlanations (SHAP), we aim not only to provide a user-friendly predictive tool but also to elucidate the contribution and interaction of key predictors, thereby facilitating personalized risk assessment and potential early intervention strategies.

## Methods

### Study design and participant

This was a single-center, retrospective, cross-sectional study. The study was conducted at the Hemodialysis Center of the Second Affiliated Hospital of Soochow University from July to December 2024, enrolling all patients receiving regular MHD during this period as the study population. The inclusion criteria were as follows: (1) age > 18 years; (2) MHD treatment duration ≥ 3 months; (3) conscious and able to cooperate in completing the questionnaire survey; and (4) voluntary provision of informed consent. Exclusion criteria included (1) actual dialysis duration < 3 months; (2) history of any malignant tumor; (3) occurrence of acute infection within the past month (e.g., respiratory or intestinal infections); (4) missing or incomplete key clinical data; and (5) inability to cooperate with the study assessments due to severe cognitive impairment or mental illness. Candidate predictors were identified from the literature on sleep disturbances in hemodialysis patients. The outcome (sleep disturbance) was objectively defined by a Pittsburgh Sleep Quality Index (PSQI) global score > 5 based on patient self-report, without assessor interpretation.

### Data collection

Data were retrospectively collected through a comprehensive review of electronic medical records and analyzed questionnaires. The collected data encompassed the following domains: (1) sociodemographic characteristics: age, sex, body mass index (BMI), education level, marital status, and economic status. (2) Clinical and dialysis-related parameters: dialysis vintage, vascular access type, dialysis frequency (sessions per week), systolic blood pressure (SBP) and diastolic blood pressure (DBP), and Kt/V. (3) Laboratory parameters: albumin (Alb), hemoglobin (Hb), blood urea nitrogen (BUN), serum creatinine (SCr), total cholesterol (TC), corrected calcium (cCa), phosphate (P), parathyroid hormone (PTH), ferritin, C-reactive protein (C-RP), and triglycerides (TG). (4) Comorbidity and etiology data: primary cause of end-stage renal disease (including chronic glomerulonephritis [CGN], hypertensive chronic kidney disease [H-CKD], diabetic kidney disease [DKD], and other etiologies), and presence of comorbidities such as diabetes, hypertension, cerebrovascular disease, and cardiovascular disease.

### Assessment of functional status, RLS, and sleep quality

Functional dependency status was assessed using both the Activities of Daily Living (ADL) scale and the Instrumental Activities of Daily Living (IADL) scale, in accordance with the Dialysis Outcomes and Practice Patterns Study (DOPPS) [16]. The Katz questionnaire [17] was employed to evaluate ADL, covering six domains: bathing, dressing, toileting, transferring, continence, and feeding. Each item is scored from 0 to 2, where 0 indicates inability to perform the activity independently and 2 indicates full independence. The total ADL score ranges from 0 to 12. The Lawton–Brody questionnaire [18] was used to assess IADL across eight domains: shopping, housekeeping, meal preparation, laundry, telephone use, transportation, managing finances, and medication management. Each item is scored from 0 (complete dependence) to a maximum score indicating full independence, with total scores ranging from 0 to 24. Based on the combined scores from both scales, participants were categorized as follows: those with an ADL score ≥ 12 and an IADL score ≥ 24 were classified as functionally independent, while those with an ADL score < 12 or an IADL score < 24 were classified as having functional dependency.

The diagnosis of RLS was established according to the standardized diagnostic criteria formulated by the International Restless Legs Syndrome Study Group (IRLSSG) [19]. These criteria encompass the essential clinical features of the condition, including an overwhelming urge to move the legs, worsening of symptoms during periods of rest or inactivity, and partial or complete relief of symptoms through movement.

Fatigue was evaluated using the SONG-HD Fatigue Scale [20], a validated instrument specifically developed for assessing fatigue in hemodialysis patients. This scale measures the severity and impact of fatigue symptoms through a standardized scoring system. In addition, post-dialysis fatigue recovery time was recorded, defined as the duration required for participants to recover from fatigue episodes following hemodialysis sessions.

Sleep quality was assessed using the validated self-reported PSQI [21]. The PSQI was used in this study solely to define the outcome variable; no sub-component analyses of the PSQI (e.g., sleep latency, sleep duration, sleep efficiency) were performed. Based on the global PSQI score, participants were categorized into two groups according to established criteria [22]: those with a total score ≤ 5 were classified as the “Normal group,” while those with a score > 5 were classified as the “Sleep Disorder group”.

### Statistical analysis

This was an exploratory model development study. The sample size was determined by all eligible patients during the study period (*n* = 208). The final sample included 144 outcome events, resulting in an events-per-variable (EPV) ratio of 28.8 for the 5 predictors in the final model, which meets the stability requirement for model development. Data analysis was performed using R software (version 4.2.2). Descriptive statistics were presented as mean ± standard deviation for normally distributed continuous variables, median (interquartile range) for non-normally distributed continuous variables, and frequency (percentage) for categorical variables. The normality of continuous variables was assessed using the Shapiro–Wilk test. Between-group comparisons (Normal group vs. Sleep Disorder group) were conducted using independent samples t-test for normally distributed continuous variables, Mann–Whitney *U* test for non-normally distributed continuous variables, and χ^2^ test or Fisher’s exact test for categorical variables, as appropriate. Spearman’s correlation analysis was employed to examine the relationships between the PSQI score and variables that showed significant differences in univariate analyses. Variables with a significance level of *P* < 0.05 in the univariate analyses were considered potential predictors and were included as candidates in a multivariable logistic regression model. To identify the most parsimonious set of independent factors associated with sleep disturbance, we employed a bidirectional stepwise selection procedure based on the Akaike Information Criterion (AIC). This approach balances model fit and complexity, allowing for both forward addition and backward elimination of variables at each step. The clinical relevance of variables was also considered during the final model selection to ensure the model’s comprehensiveness and utility in clinical practice. Specifically, although the primary cause of kidney disease as an overall variable did not reach statistical significance in every step of the algorithm (*P* > 0.05), it was retained in the final model due to its established clinical relevance and the trend observed in univariate analysis (*P* = 0.037), ensuring the nomogram provides a more comprehensive clinical risk assessment. All variables entered into the multivariable analysis were considered potential confounders based on their known or hypothesized associations with sleep disturbance from previous literature. Results were expressed as odds ratios (OR) with 95% confidence intervals (CI). A nomogram was constructed based on the final logistic regression model to predict the risk of sleep disturbance. The discriminative ability of the nomogram was evaluated using the area under the receiver operating characteristic curve (AUC). Calibration was assessed using a calibration plot, and decision curve analysis (DCA) was performed to evaluate clinical utility. To enhance the interpretability of the prediction model, we performed SHapley Additive exPlanations (SHAP) analysis on the trained eXtreme Gradient Boosting (XGBoost) model. We generated summary plots, dependency plots, and force plots to visualize the impact and contribution of each predictor at both the population and individual levels. A two-tailed P-value < 0.05 was considered statistically significant throughout the analysis.

### Ethical considerations

This study was approved by the Ethics Committee of the Second Affiliated Hospital of Soochow University (Approval No. JD-LK2024036-IR01). All procedures were performed in accordance with the ethical standards of the Declaration of Helsinki. All participants or their legally authorized representatives provided written informed consent prior to enrollment.

## Results

### Characteristics of the participants

This study enrolled 208 patients undergoing maintenance hemodialysis, of whom 144 (69.2%) were classified into the sleep disorder group based on PSQI criteria. Compared to the normal sleep group (n=64), patients with sleep disturbance were significantly older and had a higher prevalence of RLS. They also exhibited lower serum albumin levels, lower IADL scores, and altered profiles in SCr and basic ADL (all *P* < 0.05). No other significant differences were observed in a comprehensive set of sociodemographic, clinical, laboratory, and comorbidity variables (Table 1).

**Table 1:** Baseline characteristics of the normal and sleep disorder groups

Variables	Total (*n* = 208)	Normal group (*n* = 64)	Sleep disorder group (*n* = 144)	*t*/*Z*/*χ*^*2*^	*P*
Age (year)	60.14 ± 14.00	54.48 ± 14.11	62.66 ± 13.24	−4.03	< .001
Sex, *n *(%)				0.01	0.904
Female	76 (36.54)	23 (35.94)	53 (36.81)		
Male	132 (63.46)	41 (64.06)	91 (63.19)		
BMI (kg/m^2^)	22.60 ± 3.69	22.88 ± 3.71	22.47 ± 3.69	0.73	0.468
Systolic blood pressure (mmHg)	148.29 ± 18.38	145.50 ± 19.12	149.53 ± 17.97	−1.47	0.144
Diastolic blood pressure (mmHg)	78.74 ± 12.92	80.34 ± 13.56	78.03 ± 12.61	1.19	0.234
Dialysis vintage	37.00 (12.00, 59.00)	45.50 (13.00, 75.00)	33.00 (11.00, 58.00)	−1.19	0.236
Vascular access (AVF), *n*(%)				2.29	0.130
No	35 (16.83)	7 (10.94)	28 (19.44)		
Yes	173 (83.17)	57 (89.06)	116 (80.56)		
Sessions per week, *n *(%)				2.77	0.251
One	8 (3.85)	4 (6.25)	4 (2.78)		
Two	21 (10.10)	4 (6.25)	17 (11.81)		
Three	179 (86.06)	56 (87.50)	123 (85.42)		
Education, *n *(%)				0.60	0.438
≤ 9 years	135 (64.90)	44 (68.75)	91 (63.19)		
> 9 years	73 (35.10)	20 (31.25)	53 (36.81)		
Marriage, *n *(%)				2.81	0.094
No	15 (7.21)	8 (12.50)	7 (4.86)		
Yes	193 (92.79)	56 (87.50)	137 (95.14)		
Economic status (income > expenditure) *n*(%)				0.76	0.385
No	66 (31.73)	23 (35.94)	43 (29.86)		
Yes	142 (68.27)	41 (64.06)	101 (70.14)		
Albumin (g/L)	40.89 ± 3.58	42.11 ± 2.80	40.35 ± 3.76	3.74	< .001
Hemoglobin (g/L)	112.87 ± 18.09	114.34 ± 18.73	112.22 ± 17.83	0.78	0.435
Blood urea nitrogen (mmol/l)	23.74 ± 6.48	24.07 ± 6.27	23.60 ± 6.59	0.48	0.628
Serum creatinine (μmol/L)	939.83 ± 259.69	994.45 ± 268.51	915.55 ± 252.85	2.04	0.043
Kt/V	1.34 ± 0.31	1.34 ± 0.35	1.34 ± 0.29	0.05	0.963
Total cholesterol (mmol/L)	3.79 ± 0.97	3.86 ± 1.13	3.75 ± 0.89	0.75	0.452
Corrected calcium (mmol/L)	2.22 ± 0.20	2.20 ± 0.22	2.23 ± 0.20	−1.26	0.210
C-reactive protein(mg/L)	5.40 (5.10, 5.70)	5.40 (5.10, 5.62)	5.40 (5.20, 5.70)	−0.84	0.401
Triglycerides (mmol/L)	1.51 (1.10, 2.21)	1.58 (1.17, 2.48)	1.50 (1.09, 2.20)	−0.59	0.554
Phosphate (mmol/L)	1.96 (1.69, 2.30)	1.96 (1.69, 2.29)	1.96 (1.69, 2.30)	−0.50	0.618
Parathyroid hormone (pg/ml)	285.58 (178.15, 417.03)	300.16 (189.74, 485.40)	272.60 (174.87, 397.97)	−1.30	0.193
Ferritin (ng/mL)	81.95 (37.08, 167.00)	74.10 (37.32, 180.00)	84.20 (35.38, 167.00)	−0.00	0.999
Fatigue scale	3.00 (3.00, 4.00)	3.00 (3.00, 5.00)	3.00 (3.00, 4.00)	−0.32	0.748
Fatigue recovery time	2.00 (0.50, 6.00)	2.00 (0.50, 4.00)	2.00 (0.50, 8.00)	−0.70	0.486
DAL	12.00 (12.00, 12.00)	12.00 (12.00, 12.00)	12.00 (12.00, 12.00)	−2.46	0.014
IDAL	22.00 (18.00, 24.00)	24.00 (20.75, 24.00)	21.00 (15.00, 24.00)	−3.54	< .001
Primary cause, *n*(%)				7.38	0.061
CGN	122 (58.65)	45 (70.31)	77 (53.47)		
H-CKD	16 (7.69)	6 (9.38)	10 (6.94)		
DKD	42 (20.19)	8 (12.50)	34 (23.61)		
Other etiologies	28 (13.46)	5 (7.81)	23 (15.97)		
Diabetes, *n*(%)				2.19	0.139
No	148 (71.15)	50 (78.12)	98 (68.06)		
Yes	60 (28.85)	14 (21.88)	46 (31.94)		
Hypertension, *n*(%)				0.01	0.910
No	22 (10.58)	7 (10.94)	15 (10.42)		
Yes	186 (89.42)	57 (89.06)	129 (89.58)		
Cerebrovascular disease, *n*(%)				0.18	0.673
No	191 (91.83)	58 (90.62)	133 (92.36)		
Yes	17 (8.17)	6 (9.38)	11 (7.64)		
Cardiovascular disease, *n*(%)				1.26	0.261
No	189 (90.87)	56 (87.50)	133 (92.36)		
Yes	19 (9.13)	8 (12.50)	11 (7.64)		
RLS, *n*(%)				19.96	< .001
No	98 (47.12)	45 (70.31)	53 (36.81)		
Yes	110 (52.88)	19 (29.69)	91 (63.19)		

Data are presented as mean ± standard deviation, median (interquartile range), or number (percentage). Between-group comparisons were performed using the independent samples t-test, Mann–Whitney U test, χ^2^ test, or Fisher’s exact test, as appropriate

*BMI* body mass index, *DAL* activities of daily living, *IDAL* instrumental activities of daily living, *CGN* chronic glomerulonephritis, *H-CKD* hypertensive chronic kidney disease, *DKD* diabetic kidney disease, *RLS* restless legs syndrome

### Multivariable logistic regression analysis of factors associated with sleep disturbance

**Fig. 1:**
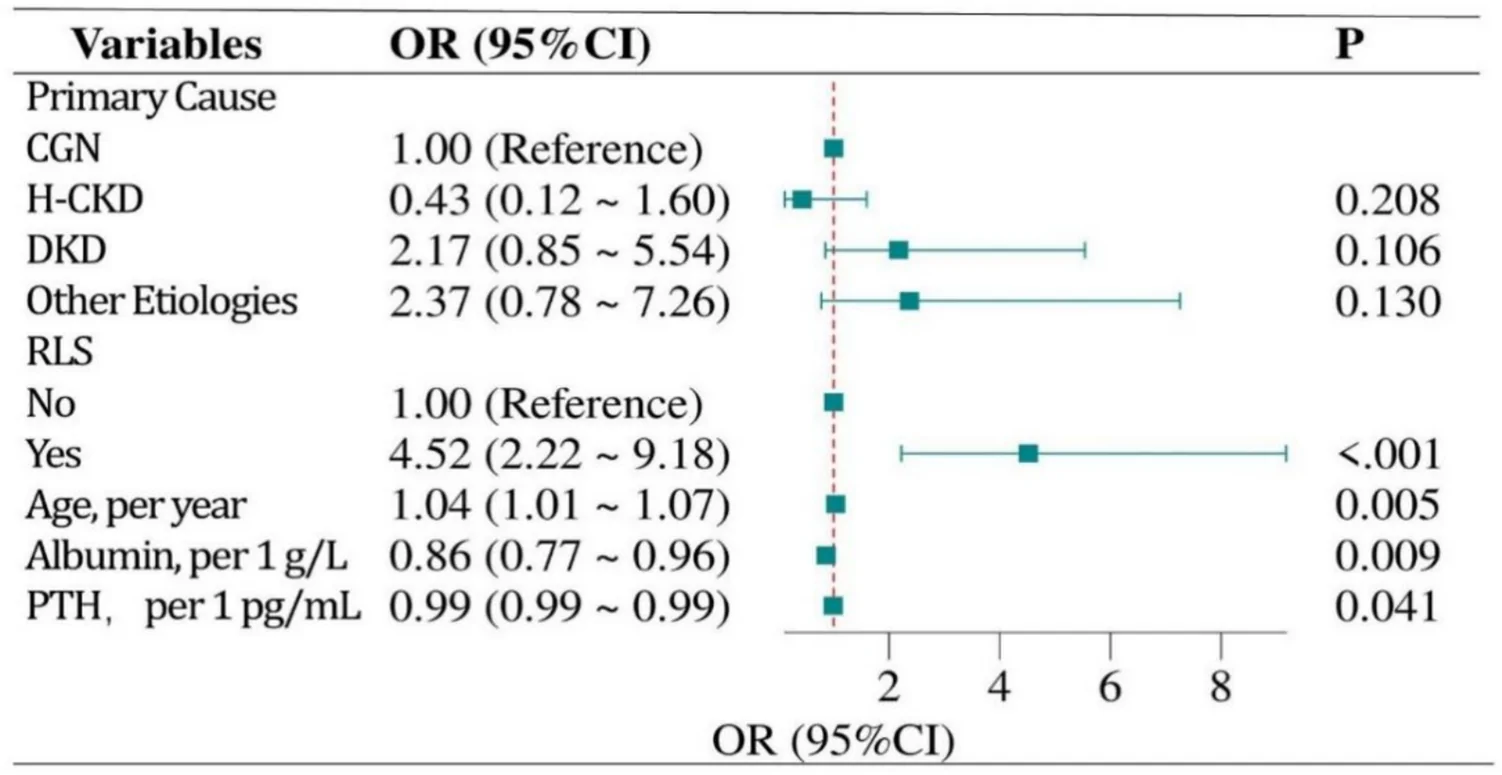
Multivariable logistic regression analysis of factors associated with sleep disturbance. Note: The model was constructed using variables with a significance level of *P* < 0.05 in the univariate analysis. *CGN* chronic glomerulonephritis, *H-CKD* hypertensive chronic kidney disease, *DKD* diabetic kidney disease, *RLS* restless legs syndrome, *PTH* parathyroid hormone

Multivariable logistic regression analysis, incorporating variables significant in the univariate analysis (*P* < 0.05), identified several independent factors associated with sleep disturbance. The presence of RLS was the strongest association, with significantly higher odds of sleep disturbance (OR = 4.52, 95% CI: 2.22–9.18, *P* < 0.001). Advanced age was also independently associated with a higher likelihood of sleep disturbance (OR = 1.04 per year, 95% CI: 1.01–1.07, *P* = 0.005). In contrast, higher serum levels of albumin (OR = 0.86 per 1 g/L, 95% CI: 0.77–0.96, *P* = 0.009) and PTH (OR = 0.99 per 1 pg/mL, 95% CI: 0.99–0.99, *P* = 0.041) were significant protective factors. Although the primary cause of kidney disease, as an overall variable, did not reach statistical significance in the multivariable model (*P* > 0.05), it was retained due to its established clinical relevance and the trend observed in univariate analysis (*P* = 0.037). This decision was made to ensure the nomogram could provide a more comprehensive clinical risk assessment. Subsequent SHAP analysis further elucidated the differential contributions of specific etiology subcategories to the predicted risk (Fig. 1).

### Receiver operating characteristic (ROC) curves for the prediction of sleep disturbance.

**Fig. 2:**
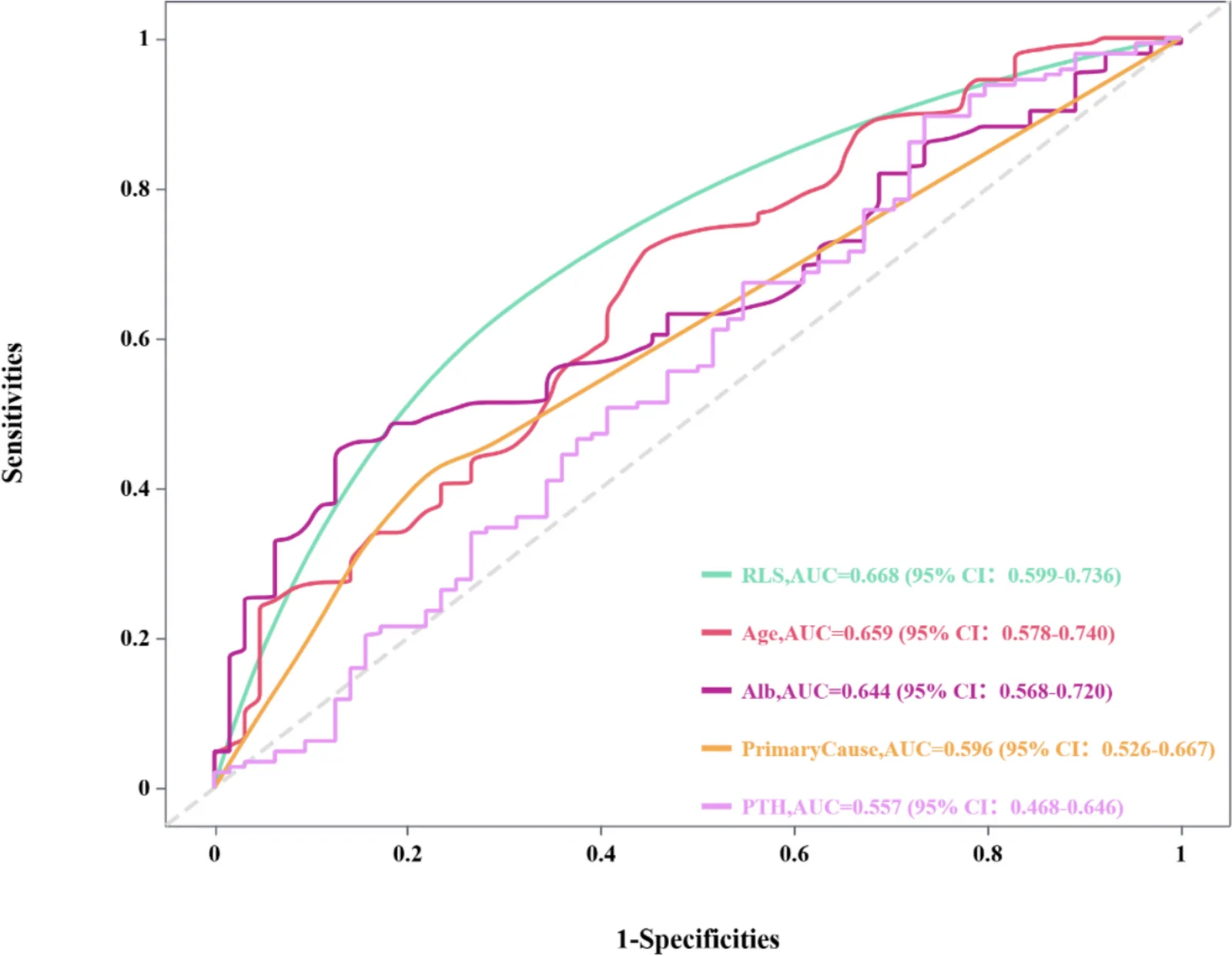
Comparison of ROC curves for variables predicting sleep disturbance. *Alb* albumin, *RLS* restless legs syndrome, *PTH* parathyroid hormone

ROC analysis revealed that RLS was the single best predictor of sleep disturbance (AUC = 0.668, 95% CI: 0.599–0.736). Age and albumin levels also demonstrated fair predictive value, with AUCs of 0.659 and 0.644, respectively (Fig. 2).

### Development of a nomogram for predicting the risk of sleep disturbance

**Fig. 3:**
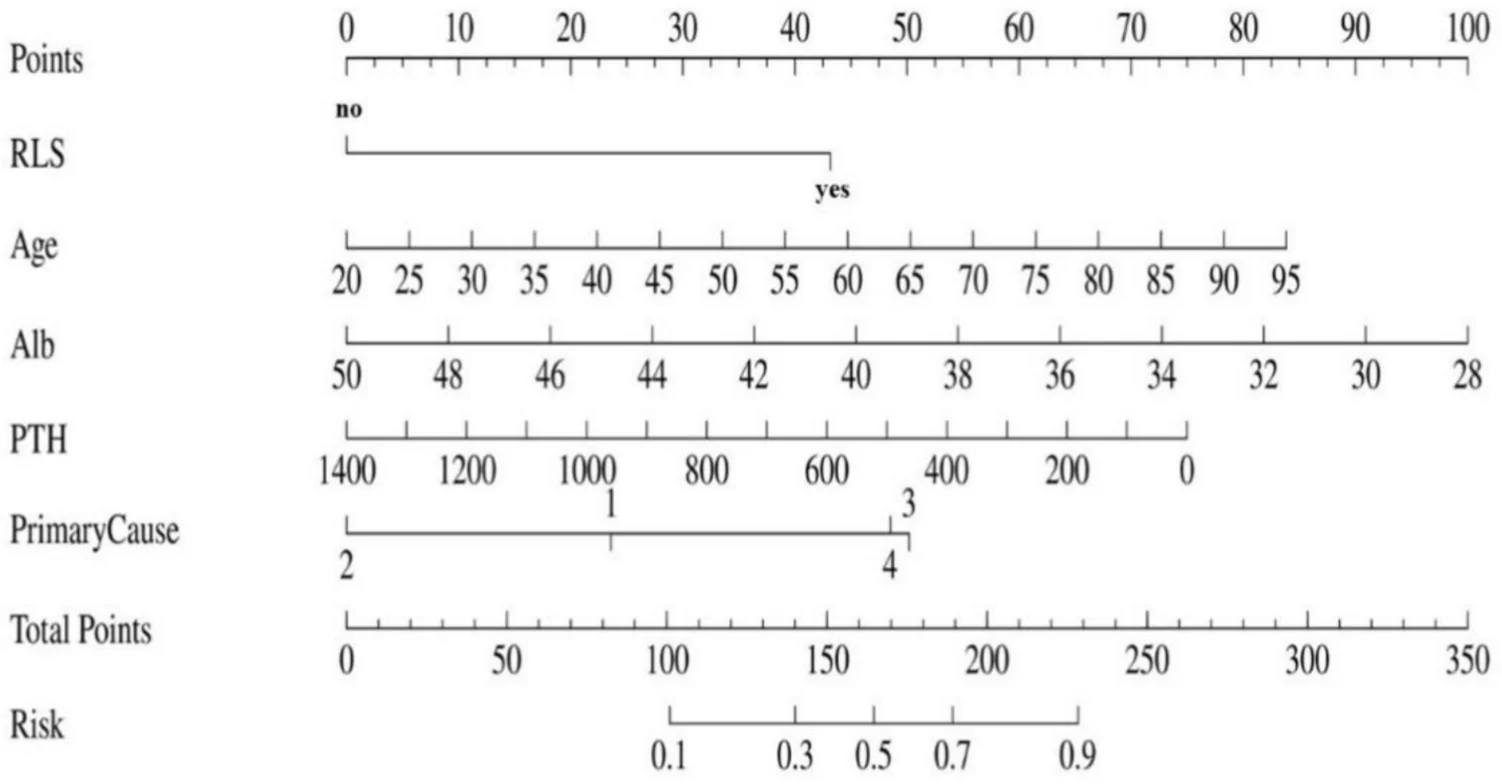
Nomogram for predicting the risk of sleep disturbance in hemodialysis patients. *Alb* albumin, *RLS* restless legs syndrome, *PTH* parathyroid hormone, primary cause: 1, chronic glomerulonephritis; 2, hypertensive chronic kidney disease; 3, diabetic kidney disease; 4, other etiologies

Based on the significant variables identified in the multivariable logistic regression analysis, a clinically applicable nomogram was developed to predict the individual risk of sleep disturbance in hemodialysis patients (Fig. 3). Recognizing that the primary etiology of kidney disease is a fundamental patient characteristic that may influence sleep pathophysiology independently of laboratory parameters, the nomogram incorporates five predictors: age, Alb, PTH, the presence of RLS, and the primary cause of kidney disease. To use the nomogram, a vertical line is drawn upward from each variable’s value to the “Points” axis. The sum of these points yields a total score, which is then projected downward to the “Total Points” and “Risk of Sleep Disorder” axes to obtain the individual’s predicted probability.

**Fig. 4:**
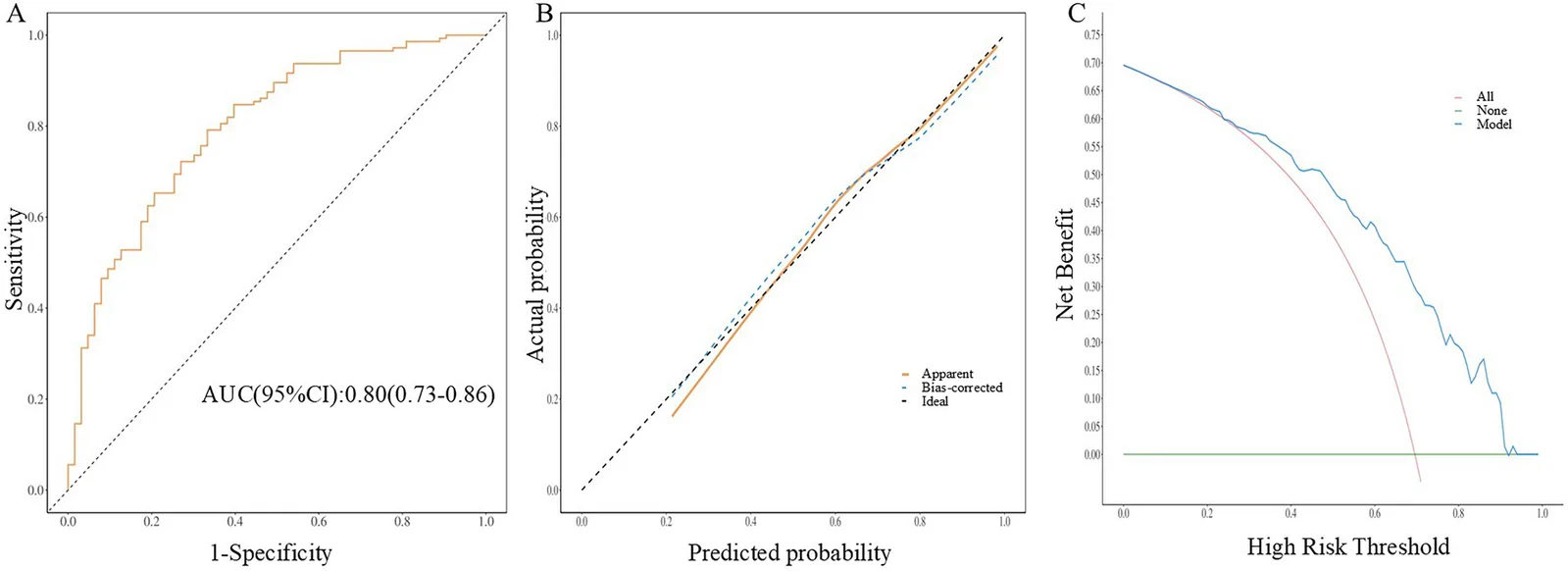
Calibration curve of the nomogram for predicting sleep disturbance risk. **A** Receiver operating characteristic (ROC) curve of the multivariable logistic regression model; **B** calibration curve of the prediction model; **C** decision curve analysis (DCA) for the prediction model

To evaluate the predictive performance of the nomogram, ROC analysis was performed, and the AUC along with other diagnostic metrics were calculated. The results showed that the nomogram had an AUC of 0.80 (95% CI: 0.73–0.86) for predicting sleep disturbance, indicating good discriminatory ability (Fig. 4A and Table 2). At the optimal cut-off value of 0.636 determined by Youden’s index, the model achieved an accuracy of 0.75 (95% CI: 0.69–0.81), a sensitivity of 0.79 (95% CI: 0.73–0.86), a specificity of 0.67 (95% CI: 0.55–0.78), a positive predictive value (PPV) of 0.84 (95% CI: 0.78–0.91), and a negative predictive value (NPV) of 0.58 (95% CI: 0.47–0.70).

**Table 2:** Performance metrics of the nomogram for predicting sleep disorders based on multivariate regression analysis

AUC (95% CI)	Accuracy (95% CI)	Sensitivity (95% CI)	Specificity (95% CI)	PPV (95% CI)	NPV (95% CI)	Cut off
0.80 (0.73–0.86)	0.75 (0.69–0.81)	0.79 (0.73–0.86)	0.67 (0.55–0.78)	0.84 (0.78–0.91)	0.58 (0.47–0.70)	0.636

Data in parentheses represent the 95% confidence interval (CI)

*AUC* area under the receiver operating characteristic curve, *PPV* positive predictive value, *NPV* negative predictive value, *cut-off* optimal prognostic threshold determined by the Youden's index. The performance metrics were derived from the multivariate logistic regression-based nomogram for predicting sleep disorders

Furthermore, internal validation of the nomogram was conducted using a calibration plot (Fig. 4B). The calibration plot demonstrated a high degree of consistency between the predicted and observed probabilities, with the curve closely aligning with the ideal 45-degree line. This visual assessment of good calibration was supported by a non-significant Hosmer–Lemeshow test (*P* = 0.95). DCA results (Fig. 4C) indicated that the use of this nomogram for predicting sleep disturbance provided a significant net clinical benefit across a wide range of probability thresholds, further supporting its potential utility in clinical practice.

### Interpreting the sleep disturbance prediction model using SHAP

**Fig. 5:**
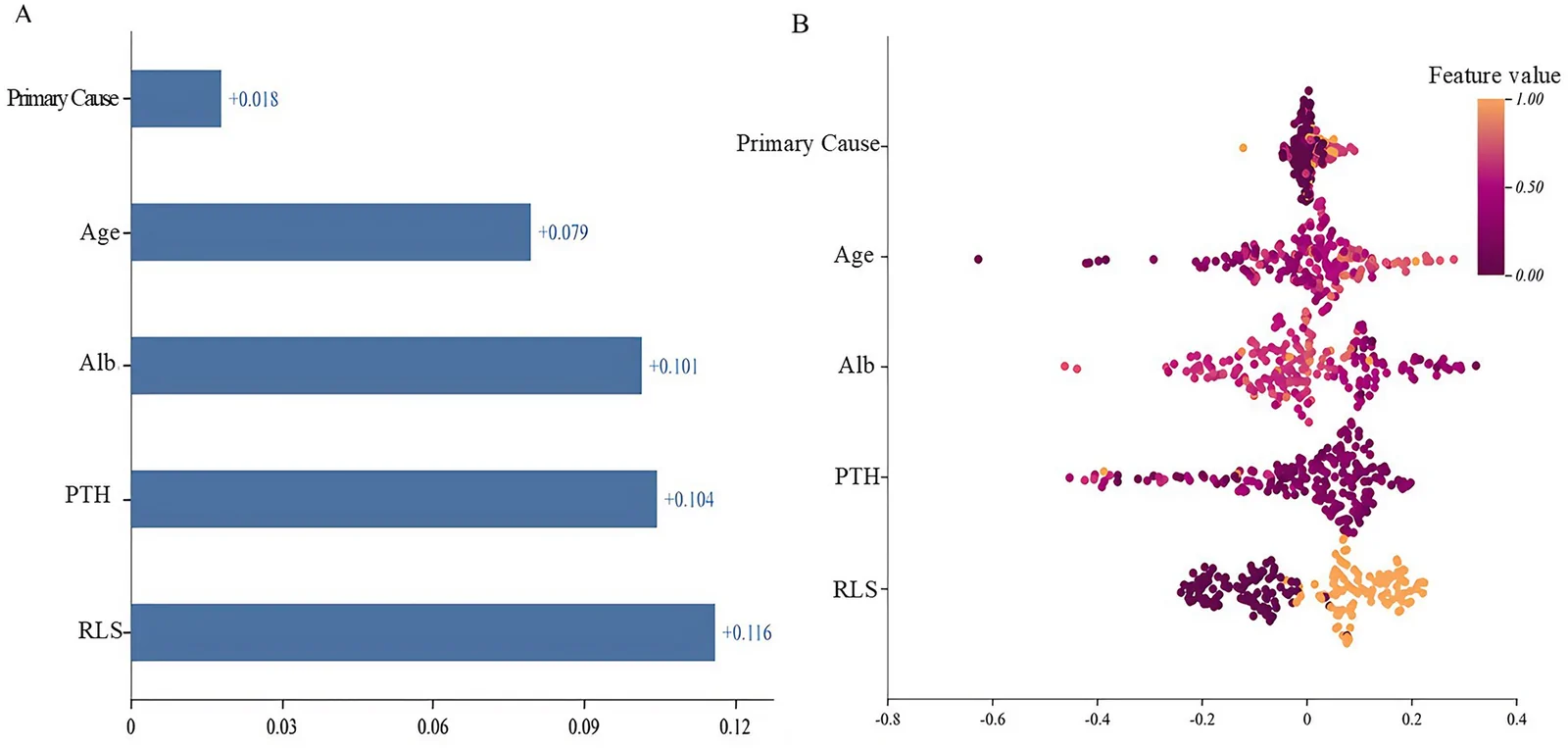
SHAP analysis for interpreting the sleep disturbance prediction model. **A **SHAP summary bar plot showing the mean absolute SHAP value for each feature, representing its overall importance in the model. **B** SHAP beeswarm plot detailing the distribution of each feature's impact on the model output for every individual in the dataset. Each point represents a patient; the color indicates the feature value (red for high, blue for low), and the horizontal position shows the effect on the prediction (positive SHAP value increases the risk of sleep disturbance). *RLS* restless legs syndrome, *Alb* albumin, *PTH* parathyroid hormone

To enhance the interpretability of the nomogram-based prediction model, we employed SHAP. The summary plot (Fig. 5A) illustrates the overall importance and impact of each feature on the model’s output. RLS was identified as the most influential predictor, followed by age, Alb, and PTH levels, which are consistent with the findings from the multivariable logistic regression analysis. The direction of the effect, represented by the color gradient, confirms that the presence of RLS and advanced age are associated with an increased risk of sleep disturbance, whereas higher levels of albumin and PTH are generally protective.

Furthermore, the SHAP beeswarm plot (Fig. 5 B) provides a detailed view of the distribution of SHAP values for each feature across all individual patients. This plot reaffirms the consistent negative impact of RLS and the positive impact of higher albumin levels. It also reveals nuanced patterns, such as the non-linear relationship between PTH and the predicted risk, which may not be captured by the logistic model alone.

### SHAP dependency plots for key predictors of sleep disturbance

**Fig. 6:**
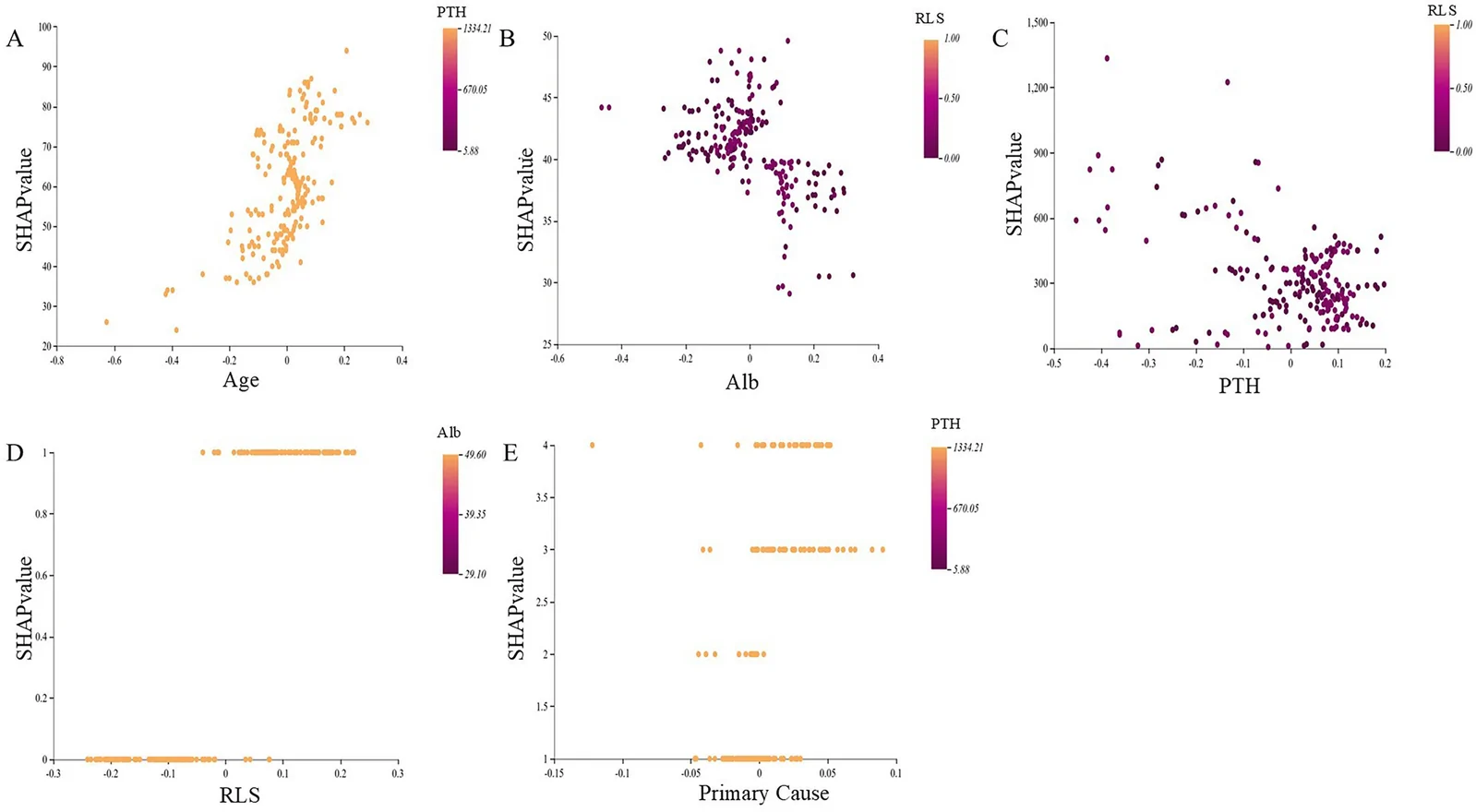
SHAP dependency plots for the key predictors in the sleep disturbance model. **A** age, **B** albumin (Alb), **C** parathyroid hormone (PTH), **D** restless legs syndrome (RLS), **E** primary cause of kidney disease. SHAP dependency plots illustrate the relationship between the value of a specific feature (x-axis) and its impact on the model prediction (SHAP value, y-axis). Each point represents a single patient observation. For continuous variables (**A**–**C**), the trend helps visualize whether the relationship is linear or non-linear. For categorical variables (**D**, **E**), the distribution of SHAP values for each category is shown. Primary cause of kidney disease categories: 1, chronic glomerulonephritis; 2, hypertensive chronic kidney disease; 3, diabetic kidney disease; 4, other etiologies

To further elucidate the functional relationship between the key predictors and the model’s output, we generated SHAP dependency plots for each of the five significant variables (Fig. 6). The plot for age (Fig. 6A) demonstrated a generally positive relationship with the predicted risk of sleep disturbance, wherein SHAP values increased with advancing age, particularly after approximately 60 years old. The dependency plot for albumin (Alb, Fig. 6B) revealed a clear negative trend, where higher serum albumin levels were associated with decreased SHAP values, indicating a protective effect against sleep disturbance. For parathyroid hormone (PTH, Fig. 6C), the relationship appeared more complex and non-linear. While very high PTH levels were associated with a slight increase in risk, there was considerable scatter, suggesting potential interaction effects or a non-uniform association. The binary nature of restless legs syndrome (RLS, Fig. 6D) was clearly captured, with the “Yes” category (presence of RLS) exhibiting markedly higher positive SHAP values compared to the “No” category, confirming its strong risk-increasing effect. The analysis for primary cause of kidney disease (Fig. 6E) suggested some variation in SHAP values across different etiologies, though the overall effect magnitude appeared modest compared to other predictors such as RLS and age.

### Force plots for individualized prediction interpretation

**Fig. 7:**
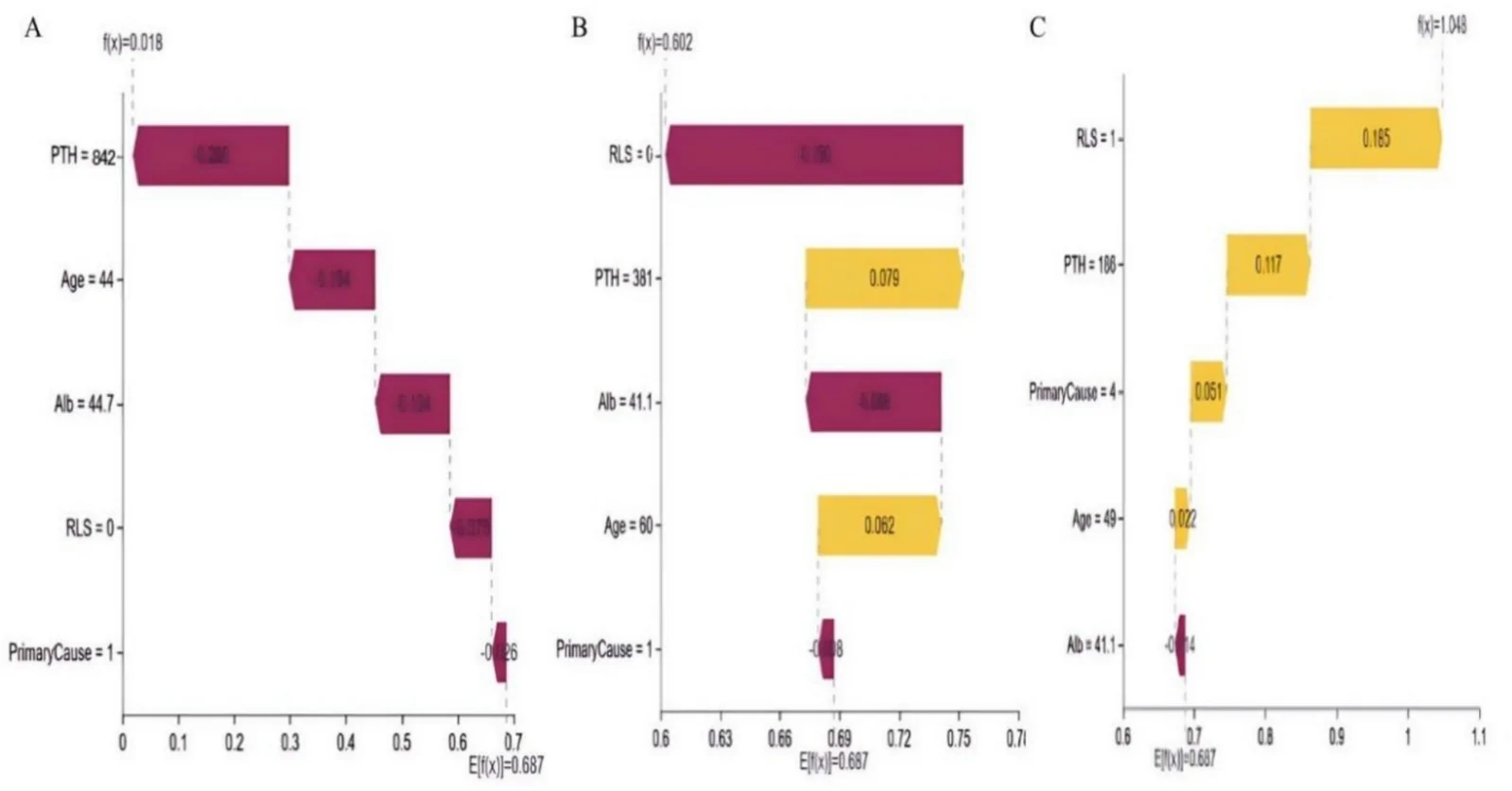
SHAP force plots illustrating individual prediction explanations for representative cases. **A** A low-risk case (final predicted probability: 1.8%). **B** A medium-risk case (final predicted probability: 60.2%). **C** A high-risk case (final predicted probability: 104.8%). The plots show how each feature value pushes the model’s base value (average prediction, E[f(X)] = 0.687) toward the final prediction (f(X)) for an individual patient. Features increasing the risk are shown in red, while those decreasing the risk are shown in blue. The length of the bar corresponds to the magnitude of the feature’s SHAP value (impact). *RLS* restless legs syndrome, *Alb* albumin, *PTH* parathyroid hormone, primary cause categories: 1, chronic glomerulonephritis; 4, other etiologies

To illustrate the individualized contribution of each predictor to the final risk score, we presented SHAP force plots for three representative cases (Fig. 7). These plots decompose how each feature drives the model’s baseline prediction (E[f(X)] = 0.687) toward the final output (f(X)) for a specific patient. In the low-risk case (Fig. 7A, f(X) = 0.018), the protective effects of younger age (44 years), higher albumin level (44.7 g/L), absence of RLS, and a specific primary cause (chronic glomerulonephritis) collectively contributed to a significant decrease in the predicted probability. Conversely, in the high-risk case (Fig. 7C, f(X) = 1.048), the presence of RLS was the strongest positive driver, followed by a specific primary cause (“Other etiologies”), and a middle-aged status (49 years), which collectively pushed the prediction well above the baseline. The medium-risk case (Fig. 7B, f(X) = 0.602) demonstrated a more balanced interplay of factors: the absence of RLS and a higher albumin level exerted protective effects, which were partially offset by an elevated PTH level and older age (60 years), resulting in a final risk score close to the baseline average.

## Discussion

This study successfully developed and validated a clinically applicable nomogram that incorporates five readily obtainable parameters—age, serum albumin, PTH, RLS, and primary kidney disease etiology—which are associated with the risk of sleep disturbance in patients undergoing MHD. The model exhibited satisfactory discriminatory power and calibration, while the implementation of SHAP methodology significantly enhanced its interpretability, offering a practical instrument for personalized risk evaluation.

Consistent with global research findings [23, 24], RLS emerged as the factor most strongly associated with sleep disturbance in our cohort. The pathophysiological basis of RLS in uremic patients—encompassing central iron deficiency, impaired dopaminergic signaling, and aberrant opioidergic neurotransmission—has been well documented in previous studies. More recent insights indicate that uremia-associated guanidino compounds may traverse the blood–brain barrier and directly interfere with dopaminergic pathways, potentially aggravating RLS manifestations [25, 26]. Our SHAP analysis quantitatively established the predominant contribution of RLS to sleep disturbance, reinforcing the clinical imperative for its systematic screening and management.

Beyond RLS, our investigation identified primary kidney disease etiology, particularly DKD, as another factor significantly associated with sleep disturbance. Even after comprehensive adjustment for covariates, DKD was independently associated with a higher likelihood of sleep disturbance compared to chronic glomerulonephritis [27]. Previous research suggests this association may be multifactorial: autonomic neuropathy in diabetes has been shown to disrupt circadian regulation through impaired melatonin secretion and thermoregulation [28]; concurrent peripheral neuropathy and cardiovascular comorbidities may promote sleep fragmentation; and glycemic instability has been reported to modulate sleep architecture via neurotransmitter alterations [29]. These observations corroborate contemporary cohort studies documenting more severe sleep impairments in diabetic nephropathy patients, underscoring the necessity for tailored sleep assessment in this subpopulation [30, 31].

Complementing these findings, we observed a clear inverse association between serum albumin levels and sleep disturbance risk, consistent with the involvement of the malnutrition-inflammation axis [32]. Hypoalbuminemia may reflect not only protein-energy wasting but also a persistent microinflammatory state. Pro-inflammatory cytokines, including IL-6 and TNF-α, have been demonstrated in experimental studies to compromise blood–brain barrier integrity and dysregulate hypothalamic–pituitary–adrenal axis function, thereby disturbing sleep–wake homeostasis [33]. Emerging evidence further suggests that albumin may modulate serotonin biosynthesis via the tryptophan–kynurenine metabolic pathway, revealing a novel mechanistic rationale for nutritional interventions in sleep management [34, 35].

In the realm of mineral metabolism, our SHAP dependency plots revealed a non-linear relationship between PTH levels and sleep disturbance risk, suggesting a complex pattern of association. While severe secondary hyperparathyroidism is conventionally viewed as detrimental to sleep [36, 37]—potentially by exacerbating uremic neuropathy and central nervous system excitation [38, 39]—our data also suggest that excessively suppressed PTH may be unfavorable. This pattern supports the concept of an optimal PTH target range for sleep health, cautioning against both extreme elevation and over-suppression in clinical management.

Furthermore, our results are consistent with the expected age-related deterioration in sleep quality reported in the literature. Beyond physiological changes such as diminished sleep spindles and reduced slow-wave sleep, elderly MHD patients may contend with polypharmacy and accelerated vascular calcification [40]. Notably, benzodiazepines and antihistamines may compound sleep architecture disruption, presenting additional therapeutic challenges [41].

It is important to acknowledge that restless legs syndrome and age emerged as the dominant predictors in our model, as evidenced by both traditional regression analysis and SHAP summary plots (Figs. 2 and 5A). This finding aligns with the well-established, profound impact of these two factors on sleep architecture in the hemodialysis population, as extensively documented in the literature [40, 42, 43]. Consequently, the overall predictive performance of the nomogram is heavily influenced by these variables. However, this does not diminish the clinical relevance of the other included factors (albumin, PTH, and primary cause). As illustrated by the SHAP force plots (Fig. 7), in individual patients, these “weaker” global predictors can still exert a substantial influence on the final risk prediction, either by mitigating or amplifying the risks posed by RLS and age. For example, in the medium-risk case (Fig. 7B), the absence of RLS exerted a protective effect, but this was partially offset by elevated PTH and older age, demonstrating the nuanced interplay among all five factors. Thus, the model’s strength lies in its ability to integrate all five factors for a personalized assessment, rather than relying on any single one. This personalized approach is precisely why we incorporated SHAP analysis—to provide transparency into how each factor contributes to individual predictions, regardless of its global importance.

Methodologically, this study represents a significant advance through its integration of conventional statistical modeling with explainable artificial intelligence. SHAP force plots enable granular visualization of variable contributions at the individual level, supporting precision clinical decision-making. Decision curve analysis confirmed the model’s net clinical benefit across a clinically relevant threshold probability range (0.2–0.8), validating its potential utility in routine practice.

### Limitations and future directions

Several limitations warrant consideration, and they impact the interpretation and generalizability of our findings. First, the single-center, retrospective, cross-sectional design not only precludes causal inference but also may introduce selection bias and limit the representativeness of our sample. As the cohort was restricted to patients from a single institution who were able and willing to participate during the study period, the possibility of selection bias cannot be excluded. This may affect the model’s generalizability to other hemodialysis populations with different demographic characteristics, clinical practices, or healthcare settings. Therefore, the findings of this study should be considered preliminary until validated in larger, more diverse, multicenter cohorts. Second, sleep assessment relied exclusively on the self-reported PSQI, which is subject to recall and reporting biases; the lack of objective measures (e.g., actigraphy) may have led to misclassification of the outcome, potentially affecting the accuracy of predictor estimates. Third, despite extensive variable inclusion, residual confounding from unmeasured factors remains possible. While variables such as diabetes mellitus, dialysis vintage, and cardiovascular disease were collected and evaluated in univariate analyses, they did not meet the statistical threshold for entry into the multivariable model. However, we acknowledge that detailed data on medications that may affect sleep or central nervous system function (e.g., hypnotics, antidepressants, antihistamines, and benzodiazepines) were not available for analysis due to the retrospective nature of this study. This represents an important unmeasured confounder that could bias the estimated associations between predictors and sleep disturbance. Future prospective studies should incorporate systematic collection of medication data to address this limitation. Finally, the absence of external validation in an independent cohort means that the reported model performance is likely optimistic, and the model’s true clinical utility and generalizability are currently unknown. In clinical application, input data should be validated for plausibility, and clear rules for handling missing key variables must be established.

To address these limitations, we recommend: (1) multicenter prospective studies for external validation and model refinement; (2) integration of objective sleep monitoring and expanded psychosocial metrics; (3) mechanistic investigations into the PTH-sleep relationship; and (4) development of targeted interventions for high-risk patients identified by the model.

## Conclusion

This study developed and validated an interpretable nomogram incorporating primary cause, RLS, albumin, PTH, and age that is associated with sleep disturbance risk in maintenance hemodialysis patients. The model demonstrated robust predictive performance and clinical utility, with SHAP analysis enhancing interpretability and revealing complex relationships between primary kidney disease etiology, mineral metabolism, and sleep outcomes. The particular vulnerability of diabetic kidney disease patients underscores the importance of etiology-specific management approaches. Future multicenter prospective studies are needed to validate this tool and explore the underlying mechanisms linking specific primary diseases to sleep pathology, ultimately facilitating its clinical translation to improve sleep quality and long-term prognosis in this population.

## Data Availability

The datasets used and analyzed during the current study are available from the corresponding author upon reasonable request. The final model object for calculating individual risk predictions is also available upon reasonable request from the corresponding author.
